# Mind-wandering rates fluctuate across the day: evidence from an experience-sampling study

**DOI:** 10.1186/s41235-018-0141-4

**Published:** 2018-12-29

**Authors:** Gabriel King Smith, Caitlin Mills, Alexandra Paxton, Kalina Christoff

**Affiliations:** 1 The University of British Columbia, Vancouver, Canada; 2 The University of New Hampshire, Durham, USA; 3 University of Connecticut, Storrs, USA

**Keywords:** Mind wandering, Freely moving thought, Experience sampling, Attention, Task-unrelated thought, Stimulus-independent thought, Daily change, Circadian rhythms

## Abstract

Previous research has demonstrated reliable fluctuations in attentional processes during the course of the day. Everyday life experience sampling, during which participants respond to “probes” delivered at random intervals throughout the day on their mobile devices, is an effective tool for capturing such diurnal fluctuations in a naturalistic way. The existence of diurnal fluctuations in the case of mind-wandering, however, has not been examined to date. We did so in two studies. In the first study, we employed everyday experience sampling to obtain self-reports from 146 university students who rated the degree of free movement in their thoughts multiple times per day over five days. These time course data were analyzed using multilevel modelling. Freely moving thought was found to fluctuate reliably over the course of the day, with lower ratings reported in the early morning and afternoon and higher ratings around midday and evening. In the second study, we replicated these effects with a reanalysis of data from a past everyday experience-sampling study. We also demonstrated differences in parameter values for the models representing freely moving thought and two common conceptualizations of mind-wandering: task-unrelated thought and stimulus-independent thought. Taken together, the present results establish and replicate a complex pattern of change over the course of the day in how freely thought moves, while also providing further evidence that freedom of movement is dissociable from other dimensions of thought such as its task-relatedness and stimulus-dependence. Future research should focus on probing possible mechanisms behind circadian fluctuations of thought dynamics.

**Electronic supplementary material:**

The online version of this article (10.1186/s41235-018-0141-4) contains supplementary material, which is available to authorized users.

## Significance

Mind-wandering has been linked to crucial parts of our daily lives, including learning, affect, and job productivity. While a number of studies have examined mind-wandering rates in everyday life, an implicit assumption has been that the rate is constant over the course of the day. The current research provides the first evidence suggesting that the extent of free movement in thought fluctuates reliably throughout our day. Thoughts appear to be most constrained in the morning and peaked in freedom of movement midday, increasing throughout the morning, decreasing throughout the afternoon, and finally increasing again in the evening. These findings may have important implications for education and the workplace, since freely moving thought could facilitate performance on tasks that require flexibility of thought (e.g. brain storming, solving novel problems), while leading to performances decrements on tasks that require stability of thought (e.g. algebra, sustained attention). Class and work schedules that adapt to the diurnal patterns of thought may lead to improved efficiency; future work should be done to explore this potential.

## Background

The last decade has seen a remarkable surge into mind-wandering research alongside its educational, clinical, and everyday life implications (Callard, Smallwood, & Margulies, 2012; Marchetti, Koster, Klinger, & Alloy, 2016; Mills, Raffaelli, Irving, Stan, & Christoff, 2017; Valdez, Ramírez, & García, 2014). Although there is no currently agreed-upon definition of mind-wandering (Christoff et al., 2018; Christoff, Irving, Fox, Spreng, & Andrews-Hanna, 2016; Irving, 2016; Seli et al., 2018; Seli, Risko, Smilek, & Schacter, 2016), there are a number of dimensions of thought that are being investigated for their presumed connection to mind-wandering. Task-unrelatedness and stimulus independence (i.e. cognition with little/no relation to external events; Schooler et al., 2011) have received the lion’s share of researchers’ attention so far (Mills, Raffaelli, et al., 2017; Seli et al., 2018). An additional dimension—freedom of movement in thought (i.e. the level of constraints on thought as it unfolds over time)—has more recently been highlighted as an important feature of mind-wandering (Christoff et al., 2016) and has been shown to be empirically dissociable from task-unrelatedness and stimulus-independence (Mills, Raffaelli, et al., 2017). One as-yet-unexplored question, however, is whether thought fluctuates systematically across the day along dimensions relevant to mind-wandering. Here, we provide the first such empirical investigation.

Until recently, a common approach in mind-wandering research was to implicitly equate mind-wandering with task-unrelated thought; however, this practice is no longer tenable due to recent developments in theoretical work on this topic (Christoff et al., 2018; Seli et al., 2018). Instead, mind-wandering is increasingly being recognized as a phenomenon that has so far evaded a one-to-one correspondence with any particular dimension of thought (Christoff et al., 2018). Although no consensus has yet been reached as to how mind-wandering relates to freedom of movement in thought, task-unrelatedness, and stimulus-independence, previous work upholds the relevance of these dimensions to understanding mind-wandering and its implications for everyday life.

In the present work, we chiefly focus on freedom of movement in thought as a dimension of thought relevant to mind-wandering and we offer (to our knowledge) the first empirical examination of its daily fluctuations (Study 1). We also examine how its diurnal fluctuations compare to the fluctuations of task-relatedness and stimulus-independence (Study 2) to present a fuller picture of the different dimensions of thought that have been linked to mind-wandering so far.

Our focus on freedom of movement stems from the potential ability of this dimension to conceptually distinguish between streams of thought that are more *constrained* (such as ruminative or goal-directed thoughts) and *spontaneously unfolding* streams of thought—an experience that is often intuitively considered to occur when the mind wanders. Within the Dynamic Framework of Thought (Christoff et al., 2016) which emphasizes how thoughts unfold over time, different sources of constraints—for example, automatic (e.g. habits, affect, salient distractors) and deliberate constraints (e.g. goal-directed focus)—dynamically influence the way thoughts unfold over time. In this model, some thought streams are more constrained (or focused on a particular topic) while other thought patterns are more “free,” i.e. they are less likely to be focused on a particular topic and will tend towards greater *content variability* over time (Mills, Herrera-Bennett, Faber, & Christoff, 2018). For example, night-time dreaming is considered to have low levels of constraint and thus higher degrees of freedom that are less bound to reality, compared to creative thinking which involves spontaneity but also elements of topical constraint; however, both can be considered forms of spontaneous thought (Christoff et al., 2016). Freedom of movement in thought would vary on a continuum from high to low based on the amount of net constraints that operate on the dynamic flow of thought at any moment, which can come from a variety of sources including (but not limited to) conscious or latent goal pursuits, habits, as well as perceptual or emotional demands.

Thoughts that are considered to have lower levels of constraint – i.e. “freely moving” thoughts – can be distinguished from other thought streams such as focused goal-directed thought and ruminative thoughts (which are influenced by imposing higher levels of constraints). Task-relatedness, despite being the most common thought dimension examined in mind-wandering research so far, does not distinguish whether a train of thought unfolds more spontaneously or in a more constrained manner. For example, rumination occurs in a constrained and fixated manner by definition (Nolen-Hoeksema, Wisco, & Lyubomirsky, 2008), which contrasts to the phenomenological experience of daydreaming; yet this contrast cannot be captured by the off-task dimension alone. Because of these distinctions, freedom of movement in thought seems to have particular relevance to mental processes in everyday life, where stimuli and tasks are much less well-defined than in an experimental context.

The majority of empirical studies on mind-wandering to date have taken place in a single session of either experience sampling in the laboratory (Franklin, Smallwood, Zedelius, Broadway, & Schooler, 2016; Krawietz, Tamplin, & Radvansky, 2012; Mills & D’Mello, 2015; Phillips, Mills, D’Mello, & Risko, 2016; Smallwood, Fitzgerald, Miles, & Phillips, 2009; Smallwood, Nind, & O’Connor, 2009; Stawarczyk, Majerus, Maj, Van der Linden, & D’Argembeau, 2011; Unsworth & McMillan, 2013) or during a single resting state functional magnetic resonance imaging (fMRI) scan (Ellamil et al., 2016; Fox, Nijeboer, Solomonova, Domhoff, & Christoff, 2013; Fox, Spreng, Ellamil, Andrews-Hanna, & Christoff, 2015; Kucyi & Davis, 2014; Mills, D’Mello, & Kopp, 2015; Phillips et al., 2016). However, because many studies are conducted between the hours of 09:00 and 17:00, they may be missing out on consistent variability in the daily cycle by collapsing results over the entire day.

The possibility that mind-wandering rates (as assessed by any of the three dimensions of thought mentioned above) may be influenced by daily cycles has been relatively ignored, despite a large body of work suggesting that time-of-day influences attention (as assessed via vigilance and selective attention tasks) as well as a wide array of cognitive faculties (Carrier & Monk, 2000; Folkard & Monk, 1987; Gabehart & Dongen, 2017). Much research in this field has been devoted to charting performance on attention-demanding cognitive tasks over the course of the day and has been successful in identifying a daily fluctuation pattern that closely parallels the rise and fall of core body temperature. Coincidentally, in parallel with the rising popularity of research on mind-wandering, analysis techniques that expand beyond simply averaging to include dynamic properties over time have also gained popularity in recent years (Allison, 2014; Dziak, Li, Tan, Shiffman, & Shiyko, 2015; Singer & Willett, 2003).

One way to address this gap in the literature is by assessing subjective ratings of thought over time in the real world: participants’ natural everyday life setting. So-called *everyday life experience-sampling* studies probe at random times throughout the day, typically using a mobile device to allow participants to answer questions (Franklin et al., 2013; Jazaieri et al., 2016; Killingsworth & Gilbert, 2010; McCormick, Rosenthal, Miller, & Maguire, 2017; McVay, Kane, & Kwapil, 2009; Song & Wang, 2012; Spronken, Holland, Figner, & Dijksterhuis, 2016). This methodology is frequently used to assess mental states over a period of time, yet longitudinal changes are often ignored in the subsequent analyses.

### The present study

Here we investigate the patterns of mind-wandering across the day in two experience-sampling studies. Study 1 focuses specifically on freely moving thought: participants were probed randomly throughout the day for five days on their cell phones as they went about their regular routines. We hypothesized that ratings of freely moving thought would significantly fluctuate throughout the day.

In Study 2, we present a new analysis of a previously published experience-sampling dataset (Mills, Raffaelli, et al., 2017) in an attempt to replicate findings from Study 1. We also ask whether different conceptualizations of mind-wandering (i.e. freely moving thought, task-unrelated thought, and stimulus-independent thought) exhibit different fluctuations over the course of the day. This idea is inspired by Mills, Raffaelli, et al.’s (2017) original finding of low overall correspondence between these distinct forms of mind-wandering; the present study, then, builds on that work to explore whether they share similar dynamics.

In the interest of open science, we have made our code and data freely available on the Open Science Framework (https://osf.io/es3gf/), and our code is additionally available on GitHub (https://github.com/galagon/mind-wandering-fluctuations).

### Study 1

#### Participants

A total of 144 participants were recruited from a large public Canadian university and were compensated for their participation with class credit. All instructions were given in English. The language of the participants was assumed to be the same: proficiency in English is an enrollment requirement for the university and only enrolled students were eligible to participate.

#### Method

All study procedures were reviewed and approved by the UBC Behavioural Research Ethics Board.

The study included an in-lab component and an experience-sampling component. First, participants underwent a 30-min training session in the lab before beginning, starting with the completion of an informed consent form and a ~ 20-min learning portion. This portion included detailed verbal instructions given via a video-recorded slideshow. The video explained the procedure, gave detailed examples for all relevant definitions, and was paused periodically to enable the experimenters to answer participants’ questions about the study and ask participants to generate novel examples of different kinds of thought. Portions of the exact script used in the video are included in Appendix 1. Finally, before leaving the lab, participants were asked to keep their phone near them at all times and respond to as many probes as possible unless it was dangerous to do so (e.g. driving).

Participants were prompted to answer probes 20 times per day for five days (yielding up to 100 total responses to probes per participant) via URL links sent directly to their mobile phones in a text message. Each probe included the question “Was your mind moving about freely?”, which was answered on a scale from *(1) Not at all* to *(7) Very much*. If it was the first probe answered that day, participants were also asked what time they had awoken that day and what time they had begun to sleep the previous night. Probes were answered from Tuesday through Saturday.

A total of 10,287 responses were collected (M = 71.4 responses per participant, SD = 21.0). Similar to Mills, Raffaelli, et al. (2017), participants who answered 60 out of 100 probes or fewer (36 participants out of 144) were dropped from the analyses to remove participants who did not follow instructions to answer sufficient thought probes for analysis. The final sample was composed of 108 participants who answered an average of 80.2 probes (SD = 14.1).

Formal and accurate *a priori* power calculations proved difficult due to the lack of information regarding effect sizes of daily changes, particularly when it came to models that incorporate multiple time terms and mixed effects as described below. In light of this, we aimed to gather as many participants as we could within a limited time window in order to maximize power, resulting in the sample size of 144 participants initially and 108 participants after exclusions. However, post-hoc observed power estimations are reported in the discussion to help with the planning of future research.

To ensure that our results were not driven by exclusion criteria, all analyses reported below were repeated with the complete dataset (i.e. no participant removal). The pattern of results remained unchanged with one exception in Study 2, noted below.

#### Data preparation

To compress the data into bins, the time at which each probe was received was rounded down to the hour and participants’ ratings of freely moving thought were averaged for each hour of the day, collapsing across different days. For some hours of the day, relatively few probe responses were logged (see Table 1 for a summary of hours with at least one probe response). Fewer than one-quarter of all participants answered a single probe during the hours of 06:00 and 07:00 (all times local and based on the 24-h clock); because of this, data pertaining to those hours were excluded from all analyses. Both Table 1 and Fig. 2 show the average freely moving thought rating for each hour of the data.

**Table 1:** Descriptive statistics of the original (Study 1) and reanalyzed (Study 2) datasets

Hour	Participants with at least one probe (n)		Freely moving thought ratings *M (SD)*	
	Study 1	Mills, Raffaelli, et al., 2017	Study 1	Mills, Raffaelli, et al., 2017
06:00	3	0	2.81 (.39)	–
07:00	24	0	3.85 (.92)	–
08:00	58	157	3.63 (.99)	3.87 (1.34)
09:00	107	161	4.05 (1.28)	3.94 (1.21)
10:00	107	165	4.17 (1.33)	4.02 (0.92)
11:00	107	165	4.19 (1.15)	4.08 (1.01)
12:00	107	165	4.26 (1.16)	4.19 (1.05)
13:00	107	165	4.30 (1.18)	4.02 (0.95)
14:00	105	165	4.19 (1.13)	4.26 (0.97)
15:00	107	164	4.18 (1.27)	4.12 (1.06)
16:00	106	165	4.03 (1.24)	4.09 (0.98
17:00	104	165	4.01 (1.15)	3.96 (1.04)
18:00	106	165	3.97 (1.31)	4.05 (1.12)
19:00	106	165	4.02 (1.19)	3.99 (1.00)
20:00	107	165	4.10 (1.33)	4.03 (1.00)
21:00	104	164	4.13 (1.30)	4.02 (1.08)
22:00	84	165	4.15 (1.41)	4.14 (1.07)
23:00	52	6	3.84 (1.47)	3.83 (1.72)

#### Approach to data analyses

Our central research question was to determine if ratings of freely moving thought fluctuate in reliable patterns over the course of the day. To answer this question, we modeled ratings of freely moving thought with the hypothesis that they would vary as a function of time. We used a mixed-effect multilevel modelling approach to test this hypothesis because: (1) it assesses change over time while preserving between-participant differences in baseline ratings (intercept) and estimated relationships (slope; Fuller-Tyszkiewicz et al., 2017); and (2) it is robust to missing data (Mirman, 2016). Below, we describe our analytical approach using current best-practices for growth curve modeling; interested readers can find more detail in Mirman (2016), Mirman, Dixon, and Magnuson (2008), and Singer and Willett (2003).

**Fig. 1:**
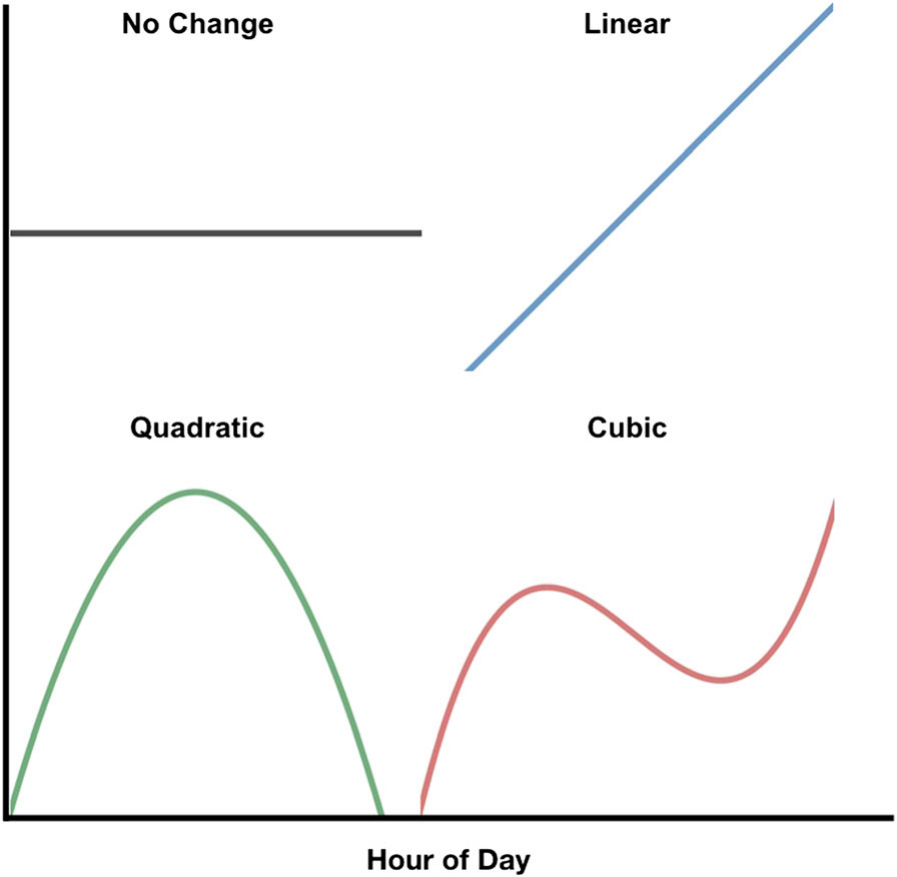
Examples of polynomial models representing change across time. The *black line* represents no change across time (intercept-only); the *blue line* represents linear change; the *green line* represents quadratic change; the *red line* represents cubic change

We tested the patterns of freely moving thought ratings by constructing a mixed-effects model with first- through third-order orthogonal polynomial terms (for comparative examples of the three orders of polynomial models; see Fig. 1). All polynomial terms were orthogonalized to avoid multicollinearity and to ensure independence from one another (see Mirman, 2016). The significance of each term would shed light on a specific pattern, though none are mutually exclusive (see Fig. 1):

1. the linear term (first order) would represent a general increase or decrease in freely moving thought over the day;
2. the quadratic term (second order) would fit the data if ratings were higher or lower in the middle of the day as compared to morning and evening; and
3. the cubic term (third order) would capture trends that changed direction twice over the day—for example, if the ratings rose throughout the morning, fell in early afternoon (i.e. first change in direction), and rose again through the evening (i.e. second change in direction).

We used polynomials to model change across time for three reasons, aside from such models being commonly used in the analysis of temporal dynamics (Hartmann et al., 2016; Mirman et al., 2008; Papageorghiou et al., 2014; Shin, 2012; White, Xie, Thompson, Loeber, & Stouthamer-Loeber, 2001). First, they can easily be orthogonalized with respect to one another, allowing us to interpret the individual contributions of each polynomial without the issue of collinearity. Second, they represent increasingly complex forms of change over time, which is important as we did not believe that simple patterns of change (e.g. linear) were likely given the literature on daily fluctuations of other cognitive variables (Burke, Scheer, Ronda, Czeisler, & Wright, 2015; Fimm & Blankenheim, 2016; Folkard & Monk, 1987; Giambra, Rosenberg, Kasper, Yee, & Sack, 1989; Goel, Basner, Rao, & Dinges, 2013; Riley, Esterman, Fortenbaugh, & DeGutis, 2017; Silva, Wang, Ronda, Wyatt, & Duffy, 2010; Valdez et al., 2005).

Finally, modeling data with these three polynomials served as a first step in understanding the dynamics of mind-wandering using some of the simplest and most commonly used forms of change found in other diurnal cognitive studies. Given the lack of previous work in this domain, we had no reason to suspect that any more complicated time-varying forms of change would provide a better fit; therefore, the choice of more specific and nuanced models would have been arbitrary. While some past studies have attempted to precisely and mechanistically model daily change in a cognitive outcome variable (Jewett & Kronauer, 1999), such models were based upon a deep and established literature that does not yet exist for freely moving thought.

#### Model construction

All analyses were performed in R (R Core Team, 2017). Models were constructed using the lme4 package (Bates, Mächler, Bolker, & Walker, 2015) and parameters were tested for significance with the lmerTest package (Kuznetsova, Brockhoff, & Christensen, 2017). All variables were centered and standardized before entry into the model, allowing us to interpret the resulting standardized estimates as effect sizes (Keith, 2005).

We constructed a model predicting freely moving thought ratings using each orthogonal polynomial as fixed effects without interaction terms. We used participants as our sole random intercept with the maximal slope structures that permitted model convergence (Barr, Levy, Scheepers, & Tily, 2013). All models were estimated using unrestricted maximum likelihood estimation, with an unstructured covariance structure.

For completeness, we also compared nested models to identify which model provided the best “fit” for the data. In this step, we constructed hierarchical models with successively higher-order time terms (i.e. intercept-only, linear, quadratic, and cubic) and tested them for improved fit over the previous iteration using chi-square tests. Ultimately, the full model (i.e. cubic model) best accounted for the data. Results from these hierarchical comparisons are reported in our supplementary materials (Additional file 1).

#### Model interpretation

We would find support for our hypothesis that ratings of freely moving thought fluctuate reliably over the course of the day if our model found significance for any time parameter (i.e. linear, quadratic, or cubic; see supplementary materials (Additional file 1) for graphical representations of each fitted model). Model parameter values can generally be interpreted as follows:

- positive linear parameters would indicate that ratings increased throughout the day;
- positive quadratic parameters would indicate that ratings were higher at the beginning and end of the day compared to the middle; and
- positive cubic parameters would indicate that ratings increased at the beginning of the day, decreased in the middle of the day, and increased again at the end of the day.

Standardization of ratings meant that the resulting coefficients of the model represent standardized (β) not raw (B) coefficients. This allows different predictors to be directly comparable (Keith, 2005). For example, if the coefficient for cubic change is higher than that for the linear change, it indicates the influence of the cubic parameter is greater or that the data conform more to a cubic form of change than a linear one.

## Results

Overall, freely moving thought ratings trended towards the center of the scale with a mean rating of 4.11 (*SD* = 1.92). As hypothesized, the results indicate that freedom-of-movement in thought did indeed fluctuate in a reliable pattern over the course of the day, as both the quadratic and cubic terms were significant. Table 2 shows the values and significance tests for each of the model parameters.

**Table 2:** Fixed effects of the optimal (cubic) model for freely moving thought ratings (original dataset)

Term	Estimate (β)	*SE*	*df*	*t* statistic	*p* value
Intercept	0.005	0.069	108.24	0.079	0.937
Linear	0.002	0.023	105.25	0.068	0.946
Quadratic	− 0.042	0.020	102.72	− 2.082	0.040^a^
Cubic	0.068	0.022	104.4	3.097	0.003^a^

*β* standardized regression coefficient

^a^Significant at α = 0.05

**Fig. 2:**
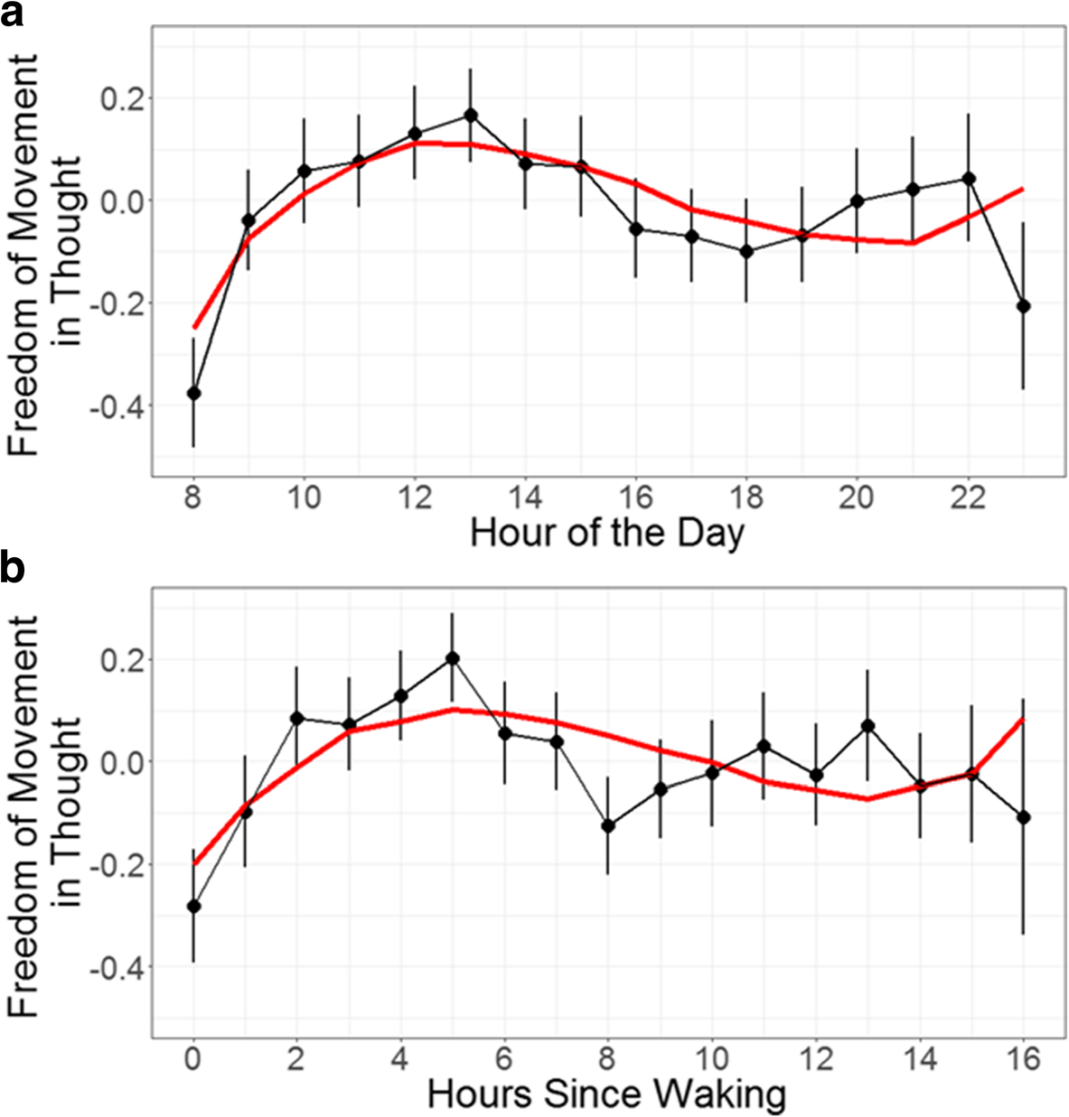
Freedom-of-movement in thought ratings (1–7) (**a**) averaged for each hour of the day and (**b**) averaged for each hour since waking. *Error bars* represent the standard error. *Red lines* represent the best-fit cubic models

Figure 2a shows this change over the course of the day. Ratings of freely moving thought were lowest in the morning (in the 08:00 h) and steadily increased until midday, before declining again. The significance of the negative quadratic term is representative of the parabolic quality of daily change in which ratings start and end low, peaking somewhere in between. The presence of the positive cubic term elaborates on this simple relationship: the peak occurs early in the day (around noon) and is followed by a decreasing pattern that changes direction and increases in the evening.

The data were also analyzed with respect to freely moving thought ratings as a function of hours awake in the day, using the self-reported wake times of participants (Fig. 2b). A similar cubic relationship was found, although the quadratic term became non-significant. Visual inspection of the form of change indicates a high degree of similarity in the two patterns, strengthening the above findings by indicating that at least the cubic pattern of change cannot be explained by variable sleep habits among participants confounding freely moving thought ratings (for full results, see Appendix 2).

## Discussion

Results from Study 1 supported our hypothesis that rates of mind-wandering change in complex patterns throughout the day. We found that participants’ thoughts were more constrained in the morning and gradually became more freely moving across the beginning of the day, peaking around noon, before falling in the afternoon and then rising again in the evening. These findings provide initial support for the idea that mind-wandering dynamically fluctuates in our everyday lives, which is important given its relationship to educational outcomes (Mills, Graesser, Risko, & D’Mello, 2017; Mrazek et al., 2017; Pachai, Acai, LoGiudice, & Kim, 2016; Seli, Wammes, Risko, & Smilek, 2016; Sousa, Carriere, & eSmilek, 2013; Valdez et al., 2014) and workplace productivity (Dane, 2011; Dust, 2015; Hyland, Lee, & Mills, 2015).

### Study 2: reanalysis of Mills, Raffaelli, et al. (2017)

Demonstrating replicability of the results from Study 1 would be highly desirable given the novelty of the research question and the exploratory nature of interpreting polynomial models assessing changes over time. Towards this goal, we conducted a novel analysis of data from Mills, Raffaelli, et al. (2017), a published experience-sampling study in everyday life that included probe timestamp information that had never been analyzed. Reanalyzing this dataset also enabled us to address the question of whether three distinct conceptualizations of mind-wandering (freely moving thought, task-unrelated thought, and stimulus-independent thought) displayed dissociable fluctuations across the day.

### Corpus

A complete description of the method from the original study can be found in Mills, Raffaelli, et al. (2017), but we provide a very brief overview here. The methodology was identical to that used in the Study 1 with four exceptions. First, the sample included 226 people initially, with 165 participants retained after removing those who answered 60% or fewer probes completely. Second, probes were given 10 times a day for a period of 10 days, yielding a maximum of 100 probes per participant (the same per-participant maximum as Study 1). Third, the questions concerning sleep and wake time were not included; instead, every probe asked participants to rate their task-unrelated thought (“Were you thinking about something other than what you were doing?”) and stimulus-independent thought (reverse-coded; “Were you aware of your surroundings?”) on a scale of 1 to 7. (Probes also included other questions that were not relevant to the present study; see Mills, Raffaelli, et al., 2017.) Fourth, participants received probes all days of the week but only between the hours of 08:00 and 23:00 (all times local and in the 24-h clock).

### Data preparation and analysis

**Fig. 3:**
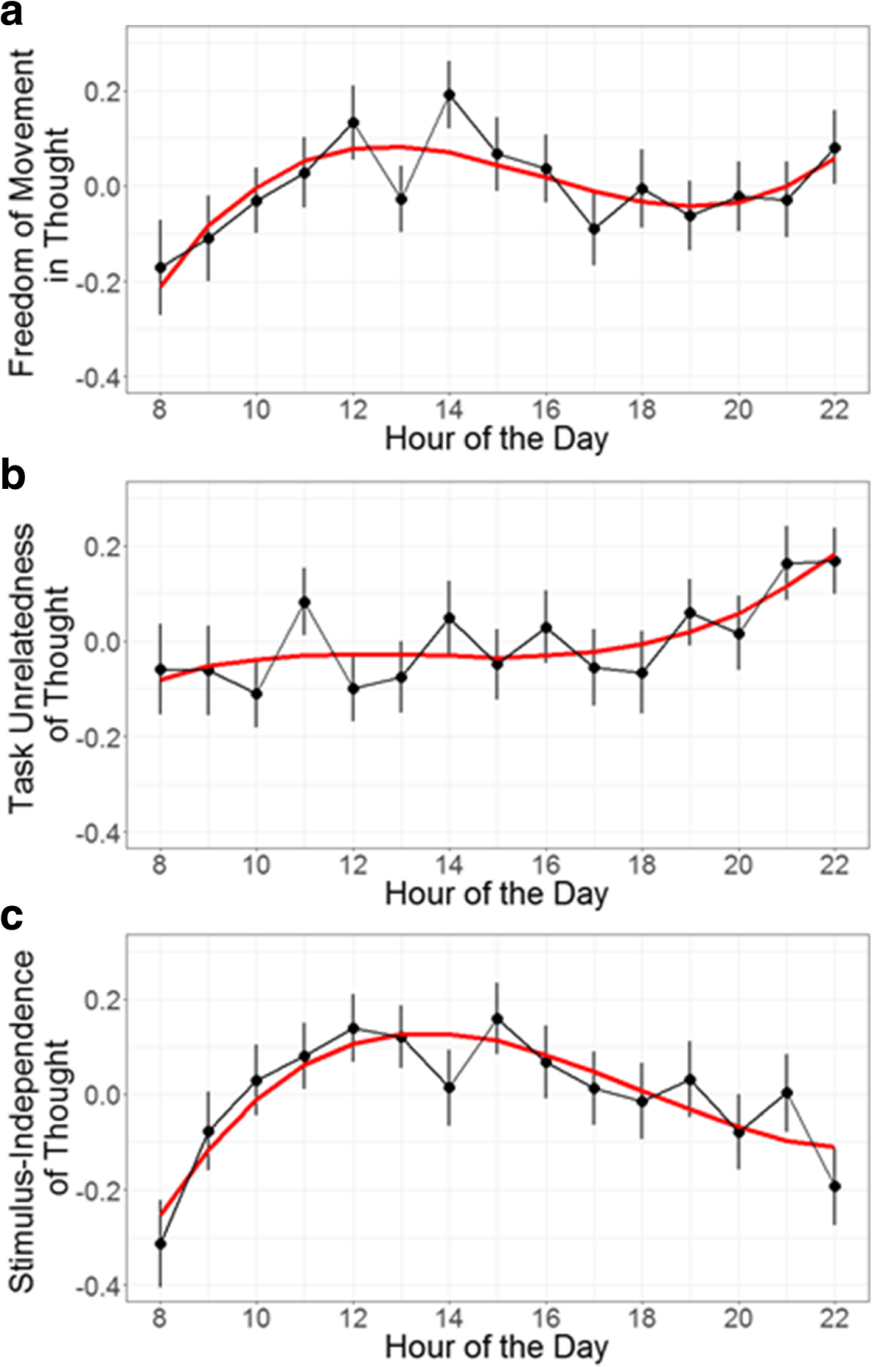
Thought dimension ratings displayed by the hour of the day. *Error bars* represent standard errors. *Red lines* represent predictions of the optimal model for each dimension (cubic in all cases): (**a**) freedom-of-movement in thought, (**b**) task-unrelatedness of thought, (**c**) stimulus-independence of thought

Similar to the analytical approach in Study 1, probes were rounded down to the hour and aggregated by person and hour. Unlike the original study, the probed hours were in the range of 08:00 to 23:00 (Mills, Raffaelli, et al., 2017). Only hour 23:00 was removed as it contained responses from < 25% of participants (Table 1). Figure 3 displays the mean ratings for each hour of the day for each dimension.

Statistical analyses were conducted with the same packages in R (lme4, Bates et al., 2015; lmerTest, Kuznetsova et al., 2017), the same fixed and random effect constraints, the same maximum likelihood effect estimation, and the same unstructured covariance structure as used with the original data. Again, all data were standardized before entry in the model, resulting in standardized (β) rather than unstandardized (B) coefficients. As with Study 1, hierarchical models were created with increasing orders of polynomial time-varying factors and compared on goodness of fit; the results of these comparisons as well as graphics representing the fitted values for each model can be found in our supplementary materials (Additional file 1).

## Results

The average rating (per participant) for each of the three definitions clustered toward the center of the scale: the mean freely moving thought rating was 4.03 (SD = 1.73), the mean task-unrelated thought rating was 4.08 (SD = 1.74), and the mean stimulus-independent thought rating was 4.19 (SD = 1.64).

We present each model’s results below. For simplicity, we do not include test statistics in the text; instead, model results are provided in Tables 3, 4, 5 and 6.

**Table 3:** Fixed effects of the optimal (cubic) model for freely moving thought ratings (reanalyzed dataset)

Term	Estimate (β)	*SE*	*df*	*t* statistic	*p* value
Intercept	− 0.001	0.052	165.0	− 0.028	0.978
Linear	0.021	0.017	166.5	1.217	0.225
Quadratic	− 0.043	0.018	171.4	− 2.409	0.017^a^
Cubic	0.059	0.017	212.2	3.435	<0.001^a^

*β* standardized regression coefficient

^a^Significant at α = 0.05

**Table 4:** Fixed effects of the optimal (cubic) model for task-unrelated thought ratings (reanalyzed dataset)

Term	Estimate (β)	*SE*	*df*	*t* statistic	*p* value
Intercept	<0.001	0.050	165.1	− 0.009	0.993
Linear	0.056	0.017	214.2	3.208	0.002^a^
Quadratic	0.027	0.017	184.2	1.565	0.119
Cubic	0.024	0.018	223.6	1.332	0.184

*β* standardized regression coefficient

^a^Significant at α = 0.05

**Table 5:** Fixed effects of the optimal (cubic) model for stimulus-independent thought ratings (reanalyzed dataset)

Term	Estimate (β)	*SE*	*df*	*t* statistic	*p* value
Intercept	<0.001	0.059	165.1	− 0.004	0.997
Linear	<0.001	0.017	162.9	− 0.018	0.986
Quadratic	− 0.099	0.015	168.4	− 6.434	<0.001^a^
Cubic	0.043	0.013	8690.6	3.130	0.002^a^

*β* standardized regression coefficient

^a^Significant at α = 0.05

**Table 6:** Fixed effects of the optimal (cubic) model for the combined model (reanalyzed dataset)

Term	Estimate (β)	*SE*	*df*	*t* statistic	*p* value
Intercept	− 0.045	0.052	165.0	− 0.857	0.393
Linear	0.022	0.015	6890.0	1.436	0.151
Quadratic	− 0.044	0.015	6890.0	− 2.924	0.003^a^
Cubic	0.060	0.015	6890.0	4.001	<0.001^a^
SI	0.121	0.083	165.0	1.467	0.144
TU	0.010	0.067	165.0	0.151	0.880
SI × Linear	− 0.023	0.021	6890.0	− 1.070	0.285
TU × Linear	0.034	0.021	6890.0	1.601	0.109
SI × Quadratic	− 0.055	0.021	6890.0	− 2.609	0.009^a^
TU × Quadratic	0.070	0.021	6890.0	3.292	0.001^a^
SI × Cubic	− 0.036	0.021	6890.0	− 0.907	0.364
TU × Cubic	− 0.148	0.021	6890.0	− 1.693	0.091

*β* standardized regression coefficient, *TU* task-unrelatedness, *SI* stimulus-independence

^a^Significant at α = 0.05

### Freely moving thought (Table 3)

Findings for freely moving thought closely replicated results from Study 1. As in the original dataset, both the quadratic and cubic terms reached significance, but the linear term did not. Notably, when the analyses were run without the removal of participants who did not answer a sufficient number of probes, the linear term became significantly positive, likely as the result of higher freedom-movement ratings in the latest hours (for more, see supplementary materials (Additional file 1).

### Task-unrelated thought

Ratings of task-unrelated thought showed linear change over the course of the day, but no other time-varying model parameters achieved significance (Table 4). The positive linear parameter indicates that the ratings increased significantly between the beginning and the end of the day. This contrasts with ratings of freely-moving thoughts, which showed quadratic and cubic change but not linear.

### Stimulus-independent thought

Ratings of stimulus-independent thought showed similar patterns of change over the day as freely moving thought, exhibiting quadratic and cubic fluctuation without linear change (Table 5).

### Comparing different dimensions of thought

Mills, Raffaelli, et al. (2017) found that freely moving thought could be distinguished from content-based definitions of mind-wandering (i.e. task-unrelated thought and stimulus-independent thought). In the present study, we provide a novel test of this claim by assessing whether these three dimensions of thought exhibit differential patterns of change across the day. Although we found that two of the dimensions (freedom-of-movement and stimulus-independence) showed similar patterns of change with the same model parameters being significant for each dimension, the individual slopes that make up the equations may be significantly different. In other words, the steepness of the curves may differ.

We tested this possibility by combining responses to all three questions in a dataset with a three-level categorical variable that we call *thought dimension* (Table 6). We then created a cubic model that included the main effect of thought dimension and its interactions with each of the three time terms. A non-significant interaction between thought dimension and a time term indicates that the given slope does not differ substantially between the three dimensions of thought, whereas a significant interaction indicates that the dimensions have dissociable slopes.

By default, the lmer function of the lme4 package in R uses treatment contrasts, meaning that it treats interactions involving categorical variables as pairwise comparisons between the first entered level of the variable (in this case, ratings of freely moving thought) and each level after that (task-unrelatedness and stimulus-independence). An analysis comparing task-unrelatedness and stimulus-independence directly can be found in the supplementary materials (Additional file 1), though we chose not to include it in the main text both to minimize the number of tables and to focus on our main research question: does freely moving thought have a distinct daily pattern compared to the dominant, content-based conceptualizations of mind-wandering?

Although there were no significant interactions between dimensions of thought and the linear or cubic terms, the results show an interaction between the quadratic term and the dimension variable with both comparisons reaching significance. The quadratic slope for ratings of freely moving thought was significantly steeper (i.e. more negative) than that of task-unrelated thought yet significantly shallower (i.e. less negative) than that of stimulus-independence thought. This pattern can be observed through visual inspection of the data (Fig. 3): freely moving thought ratings experience a rise and fall pattern over the course of the day and peak close to the middle of the day, a relationship that is exaggerated for stimulus-independent thought and absent for task-unrelated thought.

These differences in changes across the day further highlight the dissociability of the three dimensions and indicate that they may be associated with only partially overlapping neural processes or may arise from distinct constraints on agent-environment interactions.

### Weekend-only analysis

The greatest advantage of everyday life experience sampling—the fact that ratings come from participants living their daily lives and not conducting artificial laboratory tasks—comes with its own set of limitations. Without controlling the conditions under which participants answer, it is difficult to know whether the observed fluctuations are caused by intrinsic mechanisms such as diurnal rhythms or whether they are simply the product of the activities in which participants were more or less likely to be engaged in at different points in the day. For example, ratings of freely moving thought were highest around midday, a time when the study’s participants—undergraduate students—would be more likely to be in the middle of classes as compared to the evening or early morning. Aside from classes, this pattern could be driven by participants taking lunch breaks around the noon hour or, conversely, by exhaustion after long periods of intensive effort exerted in morning classes.

Although the primary goal of the study was to determine whether reliable daily fluctuations exist and not to determine their source, the reanalyzed dataset affords the opportunity to examine weekdays *and* weekend days separately. This exploratory analysis rests on the tentative assumption that daily activities on weekdays (e.g. classes, work) are scheduled differently than weekends for most participants. Thus, if daily fluctuations in thought are identical across weekdays and weekend days *despite* those days containing different sets of activities, there is evidence that activities alone may not account for these daily patterns of thought.

After removing probes answered during weekdays, 4305 of the original 12,997 probes (33.12%) remained. In order to keep the results comparable, the same participants in the full analyses were used for the weekend-only analyses, regardless of the number of probes answered after the removal of weekday probes. The analysis was limited to the reanalyzed dataset from Mills, Raffaelli, et al. (2017) due to an insufficient number of probes answered over weekend days in the dataset from Study 1.

**Fig. 4:**
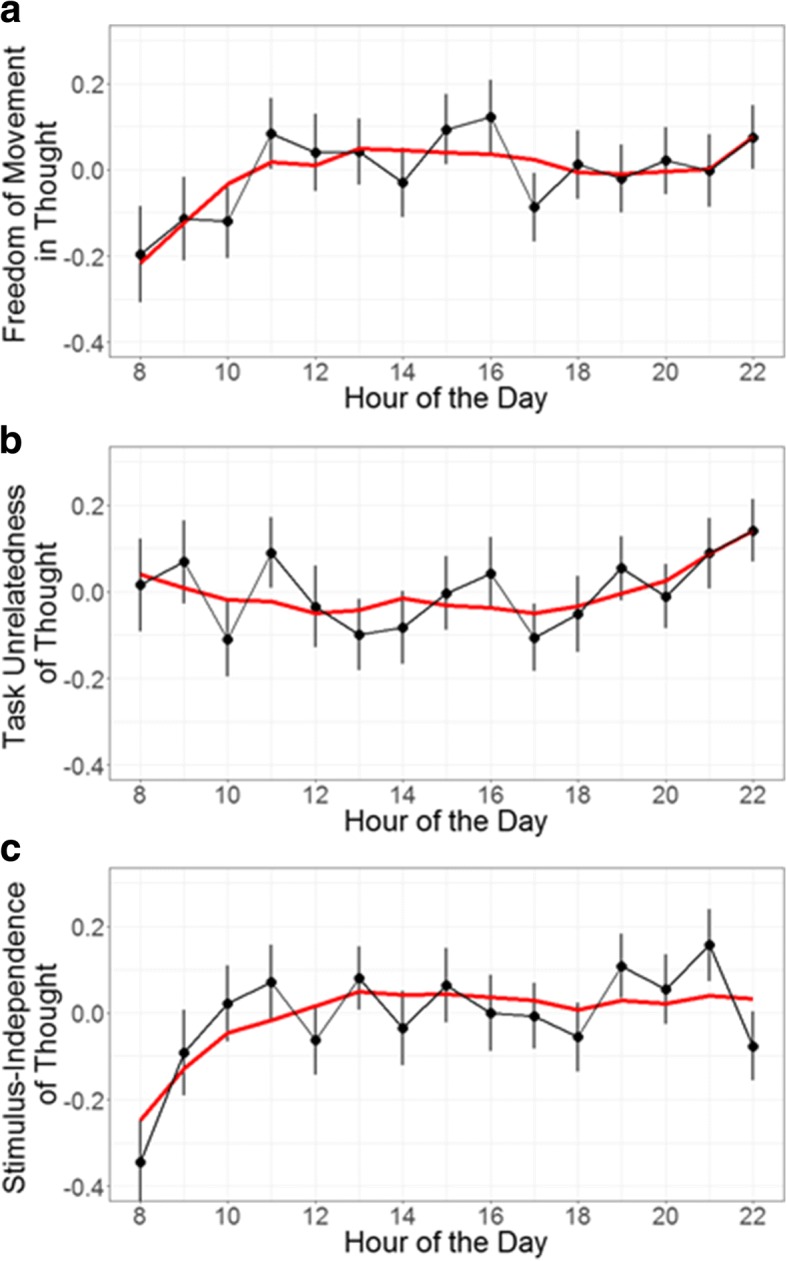
Thought dimension ratings for only weekend probes displayed by the hour of the day. Error bars represent standard errors. Red lines represent predictions of the optimal model for each dimension (cubic in all cases): (**a**) freedom-of-movement in thought, (**b**) task-unrelatedness of thought, (**c**) stimulus-independence of thought

All model results are available in Appendix 3. For brevity and clarity, we do not include most test statistics in the text, but graphical representations of the data and optimal cubic models can be seen in Fig. 4. The model for freely moving thought revealed a highly similar pattern of results, but neither the quadratic nor the cubic term quite reached statistical significance (quadratic: *p* = 0.056, cubic: *p* = 0.054; Fig. 4a). Although the lack of significance at α = 0.05 may indeed indicate a different pattern of fluctuation across the day, the loss of power accompanying a threefold reduction in the number of probes entered into the model may be an alternative reason for this disparity, especially given that the *p* values in question, while no longer significant, are still < 0.06. While we cautiously interpret the pattern for freely moving thought to be similar for the weekend-only analysis, future work with greater statistical power may be able to shed more light on this possibility.

On the other hand, we were unable to replicate the findings for the two content-based dimensions of thought. In the model predicting task-unrelatedness of thought, no time-varying parameters reached significance. In the model predicting stimulus-independence of thought, the positive linear term reached significance and the positive cubic term no longer reached significance (although it trended toward significance in the same direction), while the quadratic term remained negative and significant.

We note, however, that visual inspection of the weekend-only data indicates a highly similar pattern for task-unrelatedness of thought (Fig. 4b). This again suggests that the difference from the original analysis is possibly the result of power loss (though it is worth noting that in this case the linear term did not trend towards significance). The stimulus-independence data, on the other hand, does appear to exhibit a different relationship (Fig. 4c). Visual inspection indicates that stimulus-independence scores are higher in the evenings, which would account for the increase in the strength of the linear parameter (since the scores at the end of the day are now higher than the those at the beginning) as well as the attenuation of the cubic parameter (since the scores remain comparatively stable after midday and do not dip in the afternoon or evening).

## Discussion

The reanalysis of Mills, Raffaelli, et al. (2017) in Study 2 provided three major conclusions. First, ratings of freely moving thought followed a pattern of quadratic and cubic change across the day with an absence of linear change, replicating the findings from Study 1. This pattern was also observed *visually*—although, crucially, it failed to reach statistical significance—when analyzing only weekend data; the compatible visual and statistical trends in the data, however, suggest that the lack of statistical significance may have been due to a severe drop in statistical power by focusing only on two out of seven days of the week.

Second, two dominant, content-based dimensions of thought were also found to fluctuate across the day. Stimulus-independent thought exhibited a form of change similar to freely moving thought, while task-unrelated thought showed a simple increase in ratings throughout the day. These patterns were less reliable when analyzing weekend-only data, with stimulus-independent thought in particular appearing to exhibit a different form of daily change.

Third, the three dimensions of mind-wandering were empirically dissociable based on their quadratic parameters. Specifically, stimulus-independent thought exhibited a much stronger rise-and-fall pattern of higher scores in the midday compared to the beginning and end of the day, in comparison with freely moving thought. On the other hand, such a pattern was completely absent for task-unrelated thought.

### General discussion

Here, we presented—for the first time—evidence that thought patterns fluctuate across the day. Capturing the “wandering” mind by measuring freely moving thought, we probed people on their smartphones during their daily lives to understand the daily rhythms of thought in the “wild.” We found that freely moving thought occurs at lower rates early in the day, rises until midday, and declines gradually before rising once more in the evening. By reanalyzing a dataset from Mills, Raffaelli, et al. (2017), we were able to replicate these results and investigate novel questions about the dynamics of specific kinds of mind-wandering—in particular, we found that freely moving thought showed distinct daily patterns from two other dominant conceptualizations of mind-wandering. Such findings are consistent with a host of previous research showing that human cognition exhibits diurnal dynamics, not uniform properties over time.

### What might drive diurnal dynamics?

Although these results provide novel evidence that mind-wandering does dynamically fluctuate throughout the day, they do not speak to *why* these changes occur. We describe a few possible—but not mutually exclusive—explanations for the patterns of change. However, we recognize that much more work is required to elucidate the mechanisms driving these results, and we present these as testable hypotheses for additional future work.

The first potential mechanism is autonomic arousal. Arousal may bias attentional selectivity by way of the locus coeruleus, a remarkably well-connected norepinephrinergic brain region (Aston-Jones & Cohen, 2005; Aston-Jones & Waterhouse, 2016; Gilzenrat, Nieuwenhuis, Jepma, & Cohen, 2010; Mather, Clewett, Sakaki, & Harley, 2016). Although a direct link between arousal and the freely moving thought conceptualization of mind wandering has not been established, Mittner, Hawkins, Boekel, and Forstmann (2016) recently suggested that levels of norepinephrine originating from the locus coeruleus may differentiate between mental states that are focused on a single subject (regardless of whether the subject is the task at hand) and “exploratory,” unstable states—similar to the distinction between constrained and freely moving thought.

Indeed, diurnal fluctuations in norepinephrine align well with the pattern of results found here: an increase early in the day, a lunchtime peak, and a gradual afternoon/evening decline (Fibiger, Singer, Miller, Armstrong, & Datar, 1984; Hansen, Garde, Skovgaard, & Christensen, 2001). Previously observed fluctuations do not map perfectly onto the cubic structure of mind-wandering observed in the present dataset, given that levels of norepinephrine generally do not increase over the course of the evening. However, both share noticeable similarities throughout the rest of the day. The same pattern exists for attentional performance as well (Carrier & Monk, 2000; Silva et al., 2010; Valdez et al., 2005), suggesting that arousal may be a common mechanism behind these daily changes.

The arousal-based account may also be able to partially explain a somewhat-paradoxical finding from our study: mind-wandering rates tended to be at their *highest* during the times when past studies have indicated attentional abilities are at their peak and they tended to be at their *lowest* during the times when attentional performance is purported to suffer (Carrier & Monk, 2000; Folkard & Monk, 1987; Valdez et al., 2005). This co-fluctuation seems counter-intuitive, as the relationship between mind-wandering and performance on sustained attention tasks is often considered antithetical (McVay & Kane, 2009; Smallwood, Fitzgerald, et al., 2009; Stawarczyk et al., 2011). However, it is important to note that the majority of the studies that have found evidence of such an antithetical relationship have used task-unrelated thought as their definition of mind-wandering (oftentimes quite literally operationalized as the inverse of attentional performance); by contrast, freedom-of-movement of thought and attentional performance may not exhibit such a marked negative relationship. In light of this, the observed results would be perfectly in line with an arousal mechanism that impacts both attentional performance and thought dynamics but has no direct impact on the *content* of thought (or, at the least, not its task-relevance).

A second, simpler explanation for the observed daily fluctuations in mind-wandering may be circumstance or environmental pressure. Ratings of freely moving thought first peaked at the time of day when many participants (all of whom were students) would be in class and ratings dipped at times when they were would more likely to be home. It is therefore possible that the classroom environment may loosen constraints on thought or that other activities or states experienced at certain times of day produce the patterns seen here. Since we did not ask students to report their current activity at the time of each probe, this possibility cannot be completely discounted. However, findings for freely moving thought were partially replicated in a subset of the reanalyzed data that included only weekend days, suggesting that this pattern of change is likely independent of the day of the week, which one would not expect if it was driven by activities.

Finally, mind-wandering fluctuations may be tied to humans’ circadian rhythm in some other way. In two out of three dimensions of thought, we observed a rise-and-fall pattern that mirrors circadian body temperature changes that, in the past, have frequently been found to correlate with cognitive performance in a variety of domains (Carrier & Monk, 2000; Fimm, Brand, & Spijkers, 2016; Goel et al., 2013; Valdez, Reilly, & Waterhouse, 2008; Wright, Hull, & Czeisler, 2002). Although it would have been impractical to record temperature in an everyday life experience-sampling study, there is room for future experimental research to elaborate on the current findings by determining whether freely moving thought exhibits the same correlation with core body temperature as attention, spatial awareness, and other cognitive variables.

We recognize that our study is unable to answer these questions definitively. Perhaps some, all, or none of these explanations even work together: for example, arousal’s influence on mind-wandering could be dependent on current activity or level of motivation. As a result, we call for future work to collect the information that would be able to disentangle these effects, including concurrent measures of arousal, motivation, physiological states, and information about what participants are doing at the time of the probe responses. Such work should also continue to explore the dynamics of the three types of mind-wandering to see how, whether, and when they differ from one another.

### Implications

This paper lays the groundwork for understanding daily fluctuations in mind-wandering—a topic that may have implications for shaping real-world policy. Indeed, there is already ample evidence linking one dimension of thought related to mind-wandering (task-unrelatedness) to affect (Killingsworth & Gilbert, 2010), creativity (Baird et al., 2012), and—perhaps most compellingly for public policy—impaired comprehension and classroom performance (Mills et al., 2017; Mrazek et al., 2017; Pachai et al., 2016; Seli, Wammes, et al., 2016; Sousa et al., 2013; Valdez et al., 2014).

As a result of the link between traditional task-unrelated mind-wandering and learning, educational policy has an enormous potential to be shaped by this and other mind-wandering research. The negative association between task-unrelated thought and text comprehension has been extensively replicated (Franklin, Smallwood, & Schooler, 2011; Mrazek et al., 2017; Pachai et al., 2016; Seli, Wammes, et al., 2016; Smallwood, McSpadden, & Schooler, 2008; Sousa et al., 2013). Additional work suggests that this relationship may be partially explained by mind-wandering’s interference with students’ ability to adaptively adjust their cognitive resources to the current task demands (Mills et al., 2017).

The dynamic fluctuations in mind-wandering found here raise the question of whether students’ schedules can or should be adapted around their cognitive cycles. For example, our findings suggest that early afternoon classes may coincide with peak rates of freely moving thought and stimulus-independence—perhaps making them poor times at which to hold class. However, it is currently unclear what an optimal schedule could be, given that past research has demonstrated learning deficits in early morning classes as well due to factors such as sleepiness (see Valdez et al., 2014).

A contrasting point of view is that mind-wandering is not a hindrance to *all* educational outcomes, particularly ones that require creativity (Baird et al., 2012; Dijksterhuis & Meurs, 2006; Ellamil, Dobson, Beeman, & Christoff, 2012; Pachai et al., 2016). From this standpoint, school administrators may actually wish to structure class times to take advantage of the benefits of mind-wandering (freely moving thought, in particular), not just avoid the downsides. For example, writing and artistic classes may benefit from being held around midday, when freely moving thought is highest and constraints are lowest. Classes that require a narrower focus of attention (e.g. STEM courses) might be more effective if they are held when freely moving and task-unrelated thought are at their lowest.

It is also important to consider how daily fluctuations in thought influence performance in the workplace. Industrial-organizational research has highlighted the detrimental effects of task-unrelated thought on workplace performance (Dane, 2011;Dust, 2015 ; Hyland et al., 2015) as well as its potentially positive effects (Dust, 2015; Hyland et al., 2015). In driving simulations, task-unrelated thought has been associated variously with poorer speed control (Baldwin et al., 2017; Yanko & Spalek, 2014), slower reaction times (Yanko & Spalek, 2014), and a lack of peripheral awareness (He, Becic, Lee, & McCarley, 2011). Similarly, in real-world correlational studies, this kind of mind-wandering has been associated with risky or aggressive driving patterns (Qu et al., 2015) and responsibility for car accidents (Galéra et al., 2012). Understanding the times of day we are most likely go off-task, have unconstrained thoughts, or be unaware of our surroundings, could be a critical consideration for occupational schedules as well.

### Limitations and future directions

Of course, as an early and exploratory study, our work has a number of limitations and we see many of them are prime opportunities for future work. First, we asked people to introspectively report on their thoughts; participants’ introspective abilities have been the subject of much debate in the past (Hurlburt, Alderson-Day, Fernyhough, & Kühn, 2017; Nisbett & Wilson, 1977). However, replication of results across two independent datasets provides some evidence that individuals were responding reliably to the introspective questions. Additionally, extensive training and instructions were included during the training session to compensate for the novelty of the freely moving thought definition. Future work should strive to complement these studies by assessing the phenomenological experience of freely moving thought through its contents over time, as well as its objective signatures through physiological signals (Faber, Bixler, & D’Mello, 2018; Mills, Bixler, Wang, & D’Mello, 2016; Mills & D’Mello, 2015).

Second, we had no experimental control over participants, since the study was conducted in everyday life. This was necessary to maximize ecological validity but means that we had no way of knowing whether participants answered accurately and no way to compel them to answer rapidly. While this provided us with valuable insight into real-world processes, future work should complement naturalistic research (like the present study) with experimental designs that could shed light on processes, mediations, and causal mechanisms.

Third, in Study 1 only 71.4% of all probes were answered on average in the original dataset (80.2% after discarding low responders), leading to some variability in response rates across the hours of the day (see Table 1). This is particularly a problem for hour 23:00, as only 53 participants (fewer than half) answered a single probe during this time period, resulting in a high standard error. As a consequence, it is unclear whether the sharp drop in freely moving thought ratings from 22:00 to 23:00 represents a true effect, a problem made worse by the lack of any 23:00 probes in Study 2. We are nevertheless confident in the pattern of results leading up to 22:00 h, particularly the replicated finding that freely moving thought steadily increased throughout the morning.

Fourth, in order to ensure consistency with the existing literature, 7-point Likert scales were used to measure all three dimensions of thought. While this simplifies the process and allows participants to make quick and intuitive decisions, Likert scales have the disadvantage of being unmoored from common reference points. Put simply, even with verbal anchors, there is no objective meaning of a 4 out of 7 on the scale; as a result, each rating may mean something entirely different to each participant. Once again, we attempted to mitigate this by providing participants with extensive instructions. These instructions even included prompting participants to come up with their own examples of different levels of freely moving thought, ensuring that their ideas were as close as possible both to their cohort’s perceptions and to the experimenters’ expectations.

Fifth, past studies have found evidence that task-unrelated thought may demonstrate fluctuations (Carciofo, Du, Song, & Zhang, 2014; Giambra et al., 1989) similar to those we observed for freely moving thought or stimulus-independent thought. We did not find similar patterns for task-unrelated thought in the present study, but this may be due to significant methodological differences among the studies. Giambra et al. (1989) conducted a sleep-deprivation study intended more to explore natural circadian rhythms of mind-wandering in isolation than fluctuations in real-world environments. Because they used a lab-based task, their participants were judged on whether they were paying attention to a particular narrow assignment they were given. On the other hand, Carciofo et al. (2014) had participants estimate when they were most likely to mind-wander instead of probing them throughout the day; they used a multi-item scale that is related to but different than task-unrelated thought to judge mind-wandering. By contrast, the current study presumably assessed participants’ attention to a variety of tasks performed in their everyday life—and did so *while* they were engaged in those tasks. Taken together, it is difficult to know whether the results of this study represent a true conflict with the literature or simply a difference between experience-sampling studies and lab-based alternatives.

Finally, it is also possible that the unanswered probes differed systematically from the answered probes. Participants were instructed not to answer probes if doing so would put them in danger (e.g. if they were driving a vehicle). The fact that certain activities were un- or underrepresented may mean that ratings across the three dimensions could be biased in one direction. Of course, given the concern for participant safety, it is impossible to address this concern sufficiently in natural data; instead, experimental paradigms could induce similar settings within controlled environments (e.g. risky driving simulations used by Watson et al., 2016) to empirically test whether and how these underrepresented activities affect the dynamics.

Another important future step beyond the current work is to better understand the mechanisms that may influence the levels of freedom of movement in thought across the day. For example, how do current concerns and other conscious or latent goal pursuits influence our thought patterns, especially when affectively charged? Additionally, it may be fruitful to assess thought patterns in clinical populations that display more fixated thoughts (e.g. individuals with ruminative tendencies). Studies such as these may provide some key insights about the relationship between affect and constraints and how those features influence our freedom of movement in thought throughout our daily lives.

### Post-hoc power analyses

The lack of readily available standardized effect size estimates for mixed-effects polynomial models prevented us from determining our desired sample size based on a calculation of required statistical power. We nevertheless conducted post-hoc observed power analyses in order to inform future research about what types of sample sizes are appropriate for such effects. As observed power analyses are only conceptually meaningful when applied to tests of true effects, we conducted them only on significant parameters in order to estimate each study’s ability to detect the fixed effect in question. Although there is not consensus for how to calculate power exactly for multiple-predictor random-effects models (see Brysbaert & Stevens, 2018), we adopted a Monte Carlo simulation approach using the simR package in R (Green & MacLeod, 2015). It should be noted that due to their reliance on simulations, these numbers are not exact; however, setting a seed state before executing the analyses ensures that each analysis is replicable. Our shared code includes a fixed seed to allow readers to completely reproduce our analyses.

Table 7 lists the two significant parameters for Study 1 and the five significant parameters for Study 2, along with their corresponding observed power estimates. The sample size of 108 participants (after exclusions) in Study 1 yielded sub-optimal observed power for the quadratic parameter: 60.5% power means that if this study were replicated 100 times, over one-third of the replications would not have found the quadratic parameter to be significant. Although power for the cubic parameter was much higher (82%), future work should ideally set the sample size to ensure sufficient power for the weakest effect of interest. The sample size of 165 participants (after exclusions) used in Study 2 appears to be a little more appropriate; the power to detect the same quadratic parameter in the daily pattern of change for freely moving thought was bolstered to 67%; all other effects achieved a minimum of 80% power.

**Table 7:** Observed power for the five significant parameters of Study 2

Study	Dimension	Parameter	Observed power (%)
1	Freely moving thought	Quadratic	60.5
1	Freely moving thought	Cubic	82.0
2	Freely moving thought	Quadratic	67.0
2	Freely moving thought	Cubic	92.0
2	Task-unrelated thought	Linear	82.5
2	Stimulus-independent thought	Quadratic	100.0
2	Stimulus-independent thought	Cubic	88.0

Observed power determined through simulation as opposed to calculation; 100% power is a statistical impossibility and is the product of simulation-based estimates

In sum, although observed power was lower for Study 1 (which solely focused on freely moving thought), we are encouraged that the patterns for freely moving thought were consistent across both datasets, especially given that observed power was much higher for Study 2. Moreover, based on these analyses, similar research on this topic may want to collect > 165 participants when aiming for > 80% power to detect the smallest effect.

## Conclusion

Here, we find the first evidence that mind-wandering is not stable across the day – an important implication for both educational and occupational policy, as well as psychological research. Taken together, our findings emphasize the need to consider distinct dimensions of thought related to mind-wandering independently, a concept that has recently gained support in this research area (Mills, Raffaelli, et al., 2017; Seli, Risko, et al., 2016). A better understanding of the diurnal patterns of freely moving thought, task-unrelated thought, and stimulus-independent thought will contribute to a richer understanding of how our mental states dynamically shift over the course of our daily lives, perhaps even allowing us to optimize the structure of our days.

### Additional files

Additional file 1: **Supplementary materials. Additional file 1.** Model Comparison Results. **Additional file 2.** Complete Dataset Analyses. **Additional file 3.** Differentiation Analysis Comparing TUT and SIT. (ZIP 92 kb)

## Appendix 1

### Instructions

Notes: Every participant was trained in a small group people. The same experimenter was in the room for the entire question and answer question. The same training video was shown to all participants.

#### Introducing the study and explaining how the probes work Verbal instructions given via video

So, in this short video, we’re going to tell you everything you need to know. There’s a lot of information, so pay close attention. But we’re going to pause throughout to make sure that you’re grasping it correctly and we’re going to answer any questions. Keep those in mind. And here we go.

The basics is that we’re going to get you to rate your mental activity in a survey that you’re going to be answering a number of times throughout the day. You’re going to get text messages at random times with a web link in them, and you’re going to click that, and you’re going to go to the survey, and it’s really important that you answer right away when you get the text. Actually, you have to answer within 10 seconds. If you’re driving, or if you’re in a situation during which you absolutely can’t answer right then and there, you should just not answer at all. Don’t set it aside and try to answer later because we can see that. That being said, try to interrupt whatever you’re doing. It’s really important that you answer as many of these as possible.

Now, as soon as you hear the beep, you’re going to click on the link and go to the survey. And we’re going to get you to save the number into your phone and customize the text message tone. We’ll give you instructions later on how to do that. But it’s very important that these text messages stand out from any other text messages for reasons we’re about to tell you. You need to know right away, when you hear the tone, that it is one of these important text messages. So, when you’re answering the survey, we want you to finish within 40 seconds. Now, I mean, that may seem like a short amount of time, but once you’ve practiced a couple of times actually it’s going to get very fast. You’ll probably be able to do it within 30 seconds. So, answering quickly and intuitively is important as well. And it’s also very important that you keep your phone around you. Obviously, if you’re going to be answering quickly, and you need to answer a lot of these, keep your phone on, keep the sound up loud, and keep it within arm’s reach at all times as much as possible. And finally, you need to answer at least 80% of these probes.

So, this is a survey about your thought activity. What do we mean by thoughts? By thoughts, we mean anything that is going on in your awareness. That can include internal stuff like memories, emotions, imagining things. It can also mean external things. So, if you’re aware of sensations in your body, or things that you see or hear or smell. You get the picture.

So, when you hear that special tone on your phone, you’re going to take a mental snapshot of what’s going on in your awareness at the time. So, you hear the tone, you pause briefly, your take a snapshot, and then you’re going to open the text, click the link, and you’re going to go and answer all the questions keeping that mental snapshot in mind.

#### Explanation of free movement

Was your mind moving about freely?

Your thoughts move freely when:

- They seem to wander around, flowing from one thing to another
- There is no overarching purpose or direction to your thinking. Although there may still be some connection between one thought and the next
- Images and memories seem to spontaneously come into your mind
- Your attention lands spontaneously on things in your environment
- Your mind may spontaneously drift between things in the external environment and internal images so it may go back and forth.
- Your thoughts move freely when it feels like your thoughts could land on pretty much anything
- Or that your thoughts seem to flow with ease

For example, you know, you’re on the bus going home. You might take the following snapshot of your thoughts: You picture yourself having dinner that evening, then wonder if you’ve been eating too much fast food recently, then notice the faint music playing from another passenger’s headphones and that reminds you of a song you’ve heard at a party the night before.

This is sort of an example of your thoughts are moving freely. You know, they can also move freely around a particular topic such as a current event or, you know, something you’re currently interested in. For example, you think of the bike you just bought, then think yourself biking down a trail next weekend, then picture your friend riding next to you, then remember the first bike you got for your 10th birthday, and so on. So, this is an example where you know, it’s sharing the same topic but it’s still moving freely in that range. Thought can also move freely in the external environment. So, you might be hiking on a forest trail, you notice your mind may shift from the gravel on the path in front of you, to a slug crawling up a stump, to a leaf floating in a puddle. You understand.

So, here we’re going to pause the video, and we’re going to get you to come up with your own example of thoughts when your mind was moving about freely. The example should be different from the ones that were just mentioned.

## Appendix 2

### Freely moving thought ratings as a function of hours since waking

The same participants who had been excluded from the main analysis were excluded from the time-since-waking analysis, and an additional five of the remaining participants had no probes answered on days for which there was wake-up information, leaving a sample size of 104. Only probes answered between 0 and 16 h after waking were kept, as none of the hours after 16 contained responses from 25% or more of the sample.

The pattern of results was similar to the main analysis (Table 8), featuring a significant positive cubic parameter and no evidence of linear change over time. The main exception is the negative quadratic term, which trended toward but did not reach statistical significance. Although visual inspection of the data (Fig. 2b) suggests a highly similar pattern (with the peak occurring 5 hafter waking, dropping sharply until 8 h after waking, and rising afterward), future work should continue to tests this effect.

**Table 8:** Fixed effects of the optimal model for freely moving thought ratings by hour since waking (original dataset)

Term	Estimate (β)	*SE*	*df*	*t* statistic	*p* value
Intercept	0.001	0.066	101.1	− 0.014	<0.989
Linear	0.004	0.031	87.2	0.133	0.894
Quadratic	− 0.037	0.027	87.3	− 1.365	0.176
Cubic	0.057	0.023	96.1	2.520	0.013^a^

*β* standardized regression coefficient

^a^Significant at alpha = 0.05

## Appendix 3

### Weekend-only analyses

Full model results for the reanalysis of Mills, Raffaelli, et al. (2017) using only data points collected over weekends.

**Table 9:** Fixed effects of the optimal (cubic) model for freely moving thought ratings (reanalyzed dataset, weekends only)

Term	Estimate (β)	*SE*	*df*	*t* statistic	*p* value
Intercept	0.007	0.048	166.0	0.033	<0.001^a^
Linear	0.053	0.020	172.9	1.793	0.075
Quadratic	− 0.051	0.018	228.4	− 1.922	0.056
Cubic	0.032	0.018	176.6	1.935	0.054

*β* standardized regression coefficient

^a^Significant at alpha = 0.05

**Table 10:** Fixed effects of the optimal (cubic) model for task-unrelated thought ratings (reanalyzed dataset, weekends only)

Term	Estimate (β)	*SE*	*df*	*t* statistic	*p* value
Intercept	> − 0.001	0.042	165.2	− 0.001	>0.999
Linear	0.029	0.022	164.7	1.352	0.178
Quadratic	0.032	0.021	153.0	1.550	0.123
Cubic	0.012	0.019	156.9	0.624	0.533

*β* standardized regression coefficient

^*^Significant at alpha = 0.05

**Table 11:** Fixed effects of the optimal (cubic) model for stimulus-independent thought ratings (reanalyzed dataset, weekends only)

Term	Estimate (β)	*SE*	*df*	*t* statistic	*p* value
Intercept	0.007	0.048	166.0	0.152	0.880
Linear	0.053	0.020	172.9	2.607	0.010^a^
Quadratic	− 0.051	0.018	228.4	− 2.784	0.006^a^
Cubic	0.032	0.018	311.1	1.722	0.086

*β* standardized regression coefficient

^a^Significant at alpha = 0.05
