# Supplementary material for: Mind-wandering rates fluctuate across the day: evidence from an experience-sampling study
Source: Cogn Res Princ Implic. 2018 Dec 29;3:54. doi: 10.1186/s41235-018-0141-4 (PMC6311173; doi:10.1186/s41235-018-0141-4)
Supplement: Supplementary file 1 — Supplementary materials. Additional file 1. Model Comparison Results. Additional file 2. Complete Dataset Analyses. Additional file 3. Differentiation Analysis Comparing TUT and SIT. (ZIP 92 kb) [file 41235_2018_141_MOESM1_ESM.zip › Additional File 1.docx]

Additional File 1: Model Comparison Results

For all four data subsets (i.e., Study 1: freely-moving thought; Mills, Raffaelli, et al. [2017]: freely-moving thought, task-unrelated thought, and stimulus-independent thought), we generated successive hierarchical models to identify what model would best account for the data. The first model contained only an intercept term, and each of the following models adding a successively higher order polynomial time term (linear, then quadratic, then finally cubic). Each model was tested for whether it significantly improved data fit over the previous lower-order model (e.g., 1^st^ order vs. 0 order, then 2^nd^ order vs. 1^st^ order). Models were compared using a chi-square tests of significance with degrees of freedom equal to the additional number of parameters used.

| **Model** | ***df*** | **AIC** | **Chi-Squared (Improvement in Fit)** | **Chi-Squared *df*** | ***p* value** |
| --- | --- | --- | --- | --- | --- |
| Intercept-Only Model | 3 | 4514.8 |  |  |  |
| Linear Model | 6 | 4495.5 | 25.276 | 3 | <.001* |
| Quadratic Model | 10 | 4483.5 | 19.976 | 4 | .001* |
| Cubic | 15 | 4467.7 | 25.805 | 5 | <.001* |

*Notes*. * = significant at alpha = .05

Table S1. Model fit comparisons of hierarchical polynomial models predicting freedom of movement in thought (original data).

Figure S1. Freedom of movement in thought as a function of time of day (original data). Error bars represent standard errors. Lines are predicted values based off of fitted polynomial models (red: cubic, orange: quadratic, yellow: linear, green: intercept-only).

| **Model** | ***df*** | **AIC** | **Chi-Squared (Improvement in Fit)** | **Chi-Squared *df*** | ***p* value** |
| --- | --- | --- | --- | --- | --- |
| Intercept-Only Model | 3 | 6414.6 |  |  |  |
| Linear Model | 6 | 6413.2 | 7.4124 | 3 | .060 |
| Quadratic Model | 10 | 6399.4 | 21.7955 | 4 | <.001* |
| Cubic | 15 | 6371.1 | 38.3353 | 5 | <.001* |

*Notes*. * = significant at alpha = .05

Table S2. Model fit comparisons of hierarchical polynomial models predicting freedom of movement in thought (reanalyzed data).

Figure S2. Freedom of movement in thought as a function of time of day (reanalyzed data). Error bars represent standard errors. Lines are predicted values based off of fitted polynomial models (red: cubic, orange: quadratic, yellow: linear, green: intercept-only). Data used with permission from Mills, Raffaelli, et al., 2017.

| **Model** | ***df*** | **AIC** | **Chi-Squared (Improvement in Fit)** | **Chi-Squared *df*** | ***p* value** |
| --- | --- | --- | --- | --- | --- |
| Intercept-Only Model | 3 | 6391.1 |  |  |  |
| Linear Model | 6 | 6383.3 | 13.729 | 3 | .003* |
| Quadratic Model | 10 | 6384.9 | 6.406 | 4 | .171 |
| Cubic | 15 | 6367.9 | 27.026 | 5 | <.001* |

*Notes*. * = significant at alpha = .05

Table S3. Model fit comparisons of hierarchical polynomial models predicting task unrelatedness of thought (reanalyzed data).

Figure S3. Task unrelatedness of thought as a function of time of day (reanalysis data). Error bars represent standard errors. Lines are predicted values based off of fitted polynomial models (red: cubic, orange: quadratic, yellow: linear, green: intercept-only). Data used with permission from Mills, Raffaelli, et al., 2017.

| **Model** | ***df*** | **AIC** | **Chi-Squared (Improvement in Fit)** | **Chi-Squared *df*** | ***p* value** |
| --- | --- | --- | --- | --- | --- |
| Intercept-Only Model | 3 | 5826.9 |  |  |  |
| Linear Model | 6 | 5813.6 | 19.309 | 3 | <.001* |
| Quadratic Model | 10 | 5754.7 | 66.977 | 4 | <.001* |
| Cubic | 15 | 5750.9 | 13.748 | 5 | .017* |

*Notes*. * = significant at alpha = .05

Table S4. Model fit comparisons of hierarchical polynomial models predicting stimulus-independence of thought (reanalyzed data).

Figure S4. Stimulus independence of thought as a function of time of day (reanalysis data). Error bars represent standard errors. Lines are predicted values based off of fitted polynomial models (red: cubic, orange: quadratic, yellow: linear, green: intercept-only). Data used with permission from Mills, Raffaelli, et al., 2017.
