# Supplementary material for: Mind-wandering rates fluctuate across the day: evidence from an experience-sampling study
Source: Cogn Res Princ Implic. 2018 Dec 29;3:54. doi: 10.1186/s41235-018-0141-4 (PMC6311173; doi:10.1186/s41235-018-0141-4)
Supplement: Supplementary file 1 — Supplementary materials. Additional file 1. Model Comparison Results. Additional file 2. Complete Dataset Analyses. Additional file 3. Differentiation Analysis Comparing TUT and SIT. (ZIP 92 kb) [file 41235_2018_141_MOESM1_ESM.zip › Additional File 2.docx]

Additional File 2: Complete Dataset Analyses

Without participant removal, there were 144 participants for Study 1 and 226 for Study 2.

Table S5. Fixed effects of the optimal (cubic) model for freely-moving thought ratings (original dataset)

| **Term** | **Estimate (**β**)** | ***SE*** | ***df*** | ***t* statistic** | ***p value*** |
| --- | --- | --- | --- | --- | --- |
| Intercept | .011 | .057 | 141.96 | .193 | .847 |
| Linear | .019 | .021 | 132.05 | .930 | .354 |
| Quadratic | -.042 | .020 | 128.62 | -2.141 | .034* |
| Cubic | .068 | .020 | 130.64 | 3.488 | .001* |

*Notes*. * = significant at alpha = .05; β = standardized regression coefficient.

Figure S5. Freedom-of-movement in thought ratings (1-7) averaged for each hour of the day (original dataset, no exclusions). Error bars represent the standard error. The red line represents the best-fit cubic model.

Table S6. Fixed effects of the optimal (cubic) model for freely-moving thought ratings (reanalyzed dataset)

| **Term** | **Estimate (**β**)** | ***SE*** | ***df*** | ***t* statistic** | ***p value*** |
| --- | --- | --- | --- | --- | --- |
| Intercept | .007 | .043 | 222.80 | -.162 | .871 |
| Linear | .036 | .015 | 220.34 | 2.384 | .018* |
| Quadratic | -.041 | .016 | 243.72 | -2.599 | .010* |
| Cubic | .047 | .015 | 293.67 | 3.151 | .002* |

*Notes*. * = significant at alpha = .05; β = standardized regression coefficient.

Figure S6. Freedom-of-movement in thought ratings (1-7) averaged for each hour of the day (reanalysed dataset, no exclusions). Error bars represent the standard error. The red line represents the best-fit cubic model.

Table S7. Fixed effects of the optimal (cubic) model for task-unrelatedness ratings (reanalyzed dataset)

| **Term** | **Estimate (**β**)** | ***SE*** | ***df*** | ***t* statistic** | ***p value*** |
| --- | --- | --- | --- | --- | --- |
| Intercept | .003 | .041 | 225.80 | -.065 | .948 |
| Linear | .046 | .015 | 433.80 | 3.107 | .002* |
| Quadratic | .020 | .015 | 332.30 | 1.334 | .183 |
| Cubic | .028 | .015 | 424.00 | 1.858 | .064 |

*Notes*. * = significant at alpha = .05; β = standardized regression coefficient.

Figure S7. Task-unrelatedness in thought ratings (1-7) averaged for each hour of the day (reanalyzed dataset, no exclusions). Error bars represent the standard error. The red line represents the best-fit cubic model.

Table S8. Fixed effects of the optimal (cubic) model for stimulus-independence ratings (reanalyzed dataset)

| **Term** | **Estimate (**β**)** | ***SE*** | ***df*** | ***t* statistic** | ***p value*** |
| --- | --- | --- | --- | --- | --- |
| Intercept | -.005 | .048 | 225.9 | -.114 | .910 |
| Linear | -.007 | .015 | 210.5 | -.440 | .660 |
| Quadratic | -.098 | .014 | 207.5 | -6.869 | <.001* |
| Cubic | .040 | .012 | 105.6 | 3.248 | .001* |

*Notes*. * = significant at alpha = .05; β = standardized regression coefficient.

Figure S8. Stimulus-independence in thought ratings (1-7) averaged for each hour of the day (reanalyzed dataset, no exclusions). Error bars represent the standard error. The red line represents the best-fit cubic model.
