# Supplementary material for: Mind-wandering rates fluctuate across the day: evidence from an experience-sampling study
Source: Cogn Res Princ Implic. 2018 Dec 29;3:54. doi: 10.1186/s41235-018-0141-4 (PMC6311173; doi:10.1186/s41235-018-0141-4)
Supplement: Supplementary file 1 — Supplementary materials. Additional file 1. Model Comparison Results. Additional file 2. Complete Dataset Analyses. Additional file 3. Differentiation Analysis Comparing TUT and SIT. (ZIP 92 kb) [file 41235_2018_141_MOESM1_ESM.zip › Additional File 3.docx]

Additional File 3: Differentiation Analysis Comparing TUT and SIT

The differentiation analysis was re-run with the levels of *Dimension* reordered so that task-unrelated thought was entered first, facilitating its comparison with stimulus-independent thought. As seen in Table S8, task-unrelated thought has significantly more positive linear and quadratic parameters than stimulus-independent thought.

Table S9: Fixed Effects of the Optimal (Cubic) Model for the Combined Model (Reanalyzed Dataset).

| **Term** | | **Estimate (β)** | ***SE*** | | ***df*** | | ***t* statistic** | | ***p value*** |
| --- | --- | --- | --- | --- | --- | --- | --- | --- | --- |
| Intercept | -.034 | | .049 | 165.0 | | -.703 | | .483 | |
| Linear | .056 | | .015 | 6890.0 | | 3.700 | | <.001 | |
| Quadratic | .026 | | .015 | 6890.0 | | 1.732 | | .083 | |
| Cubic | .024 | | .015 | 6890.0 | | 1.607 | | .108 | |
| SI | | .111 | .084 | | 165.0 | | 1.325 | | .187 |
| FM | | -.010 | .067 | | 165.0 | | -.151 | | .880 |
| SI*Linear | | -.057 | .021 | | 6890.0 | | -2.671 | | .008* |
| FM*Linear | | -.034 | .021 | | 6890.0 | | -1.601 | | .109 |
| SI*Quadratic | | -.125 | .021 | | 6890.0 | | -5.901 | | <.001* |
| FM*Quadratic | | -.070 | .021 | | 6890.0 | | -3.292 | | .001* |
| SI*Cubic | | .017 | .021 | | 6890.0 | | .786 | | .432 |
| FM*Cubic | | .036 | .021 | | 6890.0 | | 1.693 | | .091 |

*Notes*. * = significant at alpha = .05; β = regression coefficient; FM = freedom of movement, SI = stimulus-independence
