# Supplementary figures and images for: Mind-wandering rates fluctuate across the day: evidence from an experience-sampling study
Source: Cogn Res Princ Implic. 2018 Dec 29;3:54. doi: 10.1186/s41235-018-0141-4 (PMC6311173; doi:10.1186/s41235-018-0141-4)

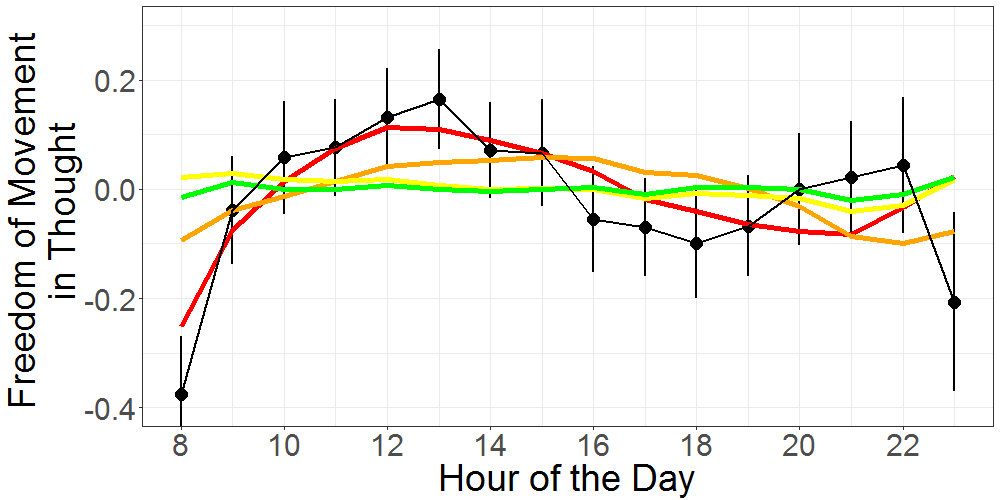

Supplement: Supplementary file 1 — Supplementary materials. Additional file 1. Model Comparison Results. Additional file 2. Complete Dataset Analyses. Additional file 3. Differentiation Analysis Comparing TUT and SIT. (ZIP 92 kb) [file 41235_2018_141_MOESM1_ESM.zip › Figure S1.png]

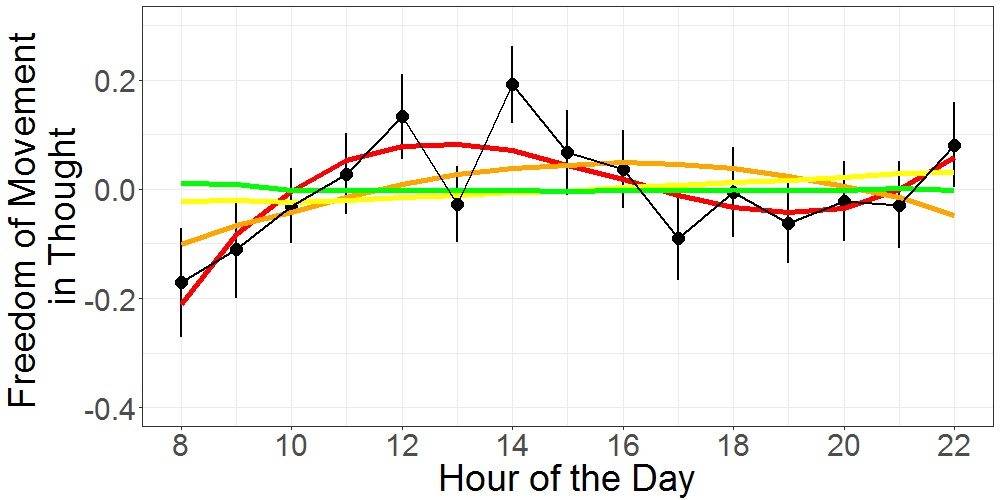

Supplement: Supplementary file 1 — Supplementary materials. Additional file 1. Model Comparison Results. Additional file 2. Complete Dataset Analyses. Additional file 3. Differentiation Analysis Comparing TUT and SIT. (ZIP 92 kb) [file 41235_2018_141_MOESM1_ESM.zip › Figure S2.png]

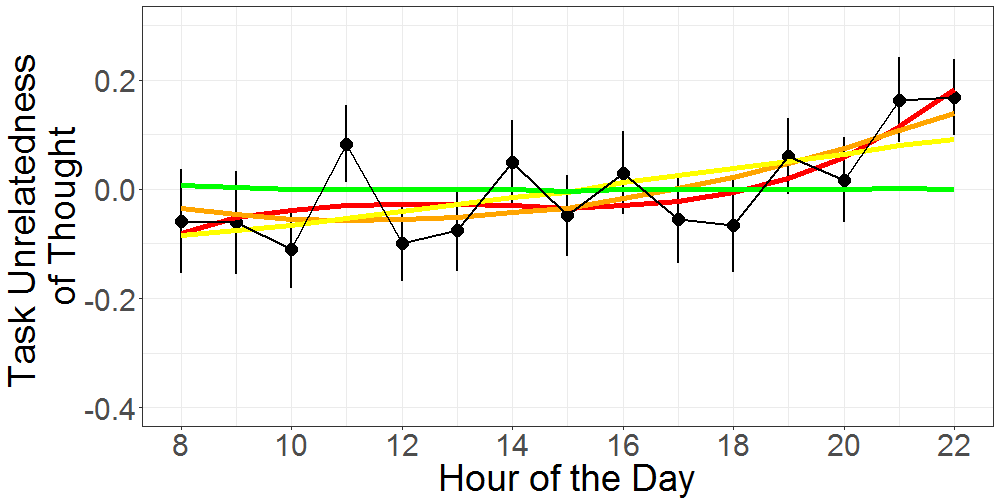

Supplement: Supplementary file 1 — Supplementary materials. Additional file 1. Model Comparison Results. Additional file 2. Complete Dataset Analyses. Additional file 3. Differentiation Analysis Comparing TUT and SIT. (ZIP 92 kb) [file 41235_2018_141_MOESM1_ESM.zip › Figure S3.png]

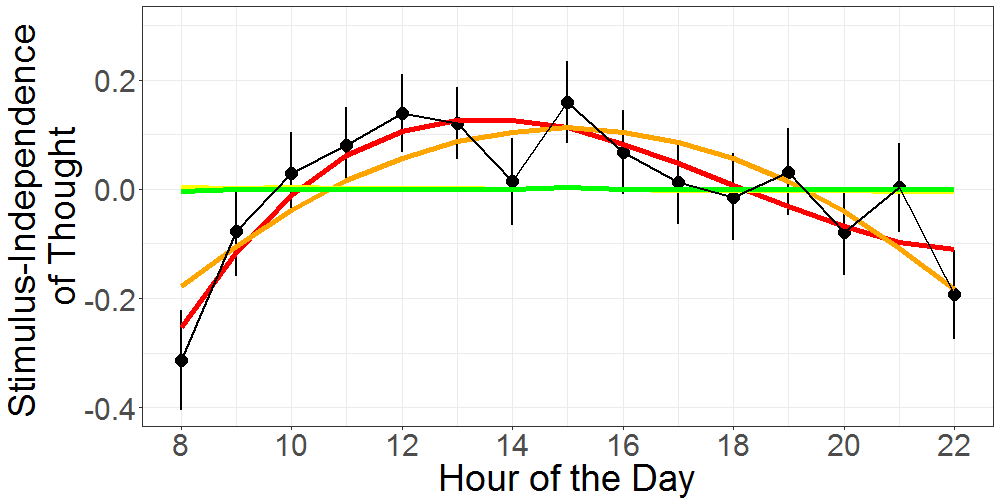

Supplement: Supplementary file 1 — Supplementary materials. Additional file 1. Model Comparison Results. Additional file 2. Complete Dataset Analyses. Additional file 3. Differentiation Analysis Comparing TUT and SIT. (ZIP 92 kb) [file 41235_2018_141_MOESM1_ESM.zip › Figure S4.png]
